# Ce(ΙΙΙ) and La(ΙΙΙ) ions adsorption through Amberlite XAD-7 resin impregnated via CYANEX-272 extractant

**DOI:** 10.1038/s41598-023-34140-9

**Published:** 2023-04-28

**Authors:** Azadeh Yarahmadi, Mohammad Hassan Khani, Masoud Nasiri Zarandi, Younes amini

**Affiliations:** 1 Department of Chemical Engineering, Faculty of Oil and Gas Engineering, Semnan University, P.O.BOX: 35131-1911, Semnan, Iran; 2 Nuclear Fuel Cycle Research School, Nuclear Science and Technology Research Institute, P.O.BOX: 11365-8486, Tehran, Iran

**Keywords:** Environmental sciences, Chemistry, Engineering

## Abstract

The goal of this paper is to investigate the ability of Amberlite XAD-7 (AXAD-7) resin impregnated with CYANEX-272 (di-2,4,4-trimethylpentyl phosphonic acid) to remove cerium (Ce(ΙΙΙ)) and lanthanum (La(ΙΙΙ)) ions from aqueous solutions in the batch scheme. The prepared adsorbent material was determined utilizing FTIR, SEM–EDX, and BET methods. The impact of three individual process variable factors involving feed solution pH (2–6), adsorbent dose (0.05–0.65), and process temperature (15–55 °C) on the simultaneous removal of Ce(ΙΙΙ) and La(ΙΙΙ) ions was evaluated via response surface methodology (RSM) according to the central composite design (CCD). The modeling of Ce(ΙΙΙ) and La(ΙΙΙ) ions adsorption was performed using the quadratic model and was evaluated using a coefficient of determination for both ions. The optimization data revealed that the adsorption amount of Ce(ΙΙΙ) and La(ΙΙΙ) ions removal under optimal conditions were 99.75% and 69.98%, respectively. Equilibrium and kinetic investigations were also conducted to define the removal performance of the calculated adsorbent for Ce(ΙΙΙ) and La(ΙΙΙ) ions removal. Various isotherms models such as Langmuir, Freundlich, Temkin, and Sips were examined at 25 °C to analyze the equilibrium isotherm data. The data revealed that the Sips approach is compatible with the experimental data. The highest adsorption capacity of the resin for Ce(ΙΙΙ) and La(ΙΙΙ) ions were 11.873 mg g^−1^ and 7.324 mg g^−1^, correspondingly. The kinetic study of the Ce(ΙΙΙ) and La(ΙΙΙ) adsorption process was conducted via pseudo-first-order, pseudo-second-order, and intraparticle diffusion models(IDMs). Based on the data obtained, kinetic data were fitted well to a pseudo-second-order rate correlation. According to the obtained results, the (AXAD-7) resin impregnated with CYANEX-272 performed well in removing both Ce(ΙΙΙ) and La(ΙΙΙ) ions from aqueous solutions with well stability during several adsorption–desorption cycles and well regeneration and excellent metallic ions recovery.

## Introduction

Rare earth elements (REE), which are in the category of industrial, chemical, and precious metals, contain 15 elements of lanthanides from lanthanum(57) to lutetium(71) and the two components of yttrium (39) and scandium (21)^1–4^. (REE)have similar physical and chemical properties due to their stable trivalent ions of the same size. The unique properties of these elements have caused them to be widely used in various industries in recent decades, such as the nuclear industry, ceramic, and glass production^5–8^, petrochemical and catalytic industry^9,10^, electronics and metallurgy, etc.^11,12^. Amongst the REE, cerium (Ce) and lanthanum (La) have received growing interest recently, due to their high technological applications^13–16^.

Due to the wide applications of these Rare earth elements in new industries, key technologies, and their commercial importance, their demand is remarkably increased^17^. There is a high probability that a significant amount of the cerium and lanthanum elements will enter the environment during their separation and purification process. These elements collect in the human body when inhaled or digested from the food chain and could generate several problems for human health^18^. Common approaches for the elimination of REE from aqueous solutions involve reverse osmosis, solvent extraction^19^, chemical deposition, ion exchange, adsorption, etc.^20^. Among these various methods, the adsorption process using solvent-impregnated resins (SIR) demonstrated an efficient, effective, and economical technology^21^. In this method, the surface of the resin is impregnated with a chelating solvent, usually containing the organic compounds of the cavity, and the ion exchange process is performed by it^22^. SIRs have the properties of ion exchange resins and solvent extraction methods simultaneously^19,23^. Compared to other separation methods, this technology has advantages such as simple design, more flexibility, suitable physical and chemical stability, production of minimal secondary residues, and the possibility of resin recovery^24^. Resin surface properties and extractant functional groups are important in SIR efficiency and the adsorption process^25^. XAD series microporous resins are one of the most important resins in the preparation of SIR and as a neutral polymer base; having a strong structure with a highly effective level to provide the best substrate for SIR^26^. So far the adsorption of many metals such as Cu(II), Fe(II), Pb(II), Cd(II), Zn(II), Bi(III), and Co(II) has been successfully performed using SIRs by the selection of suitable extractant^27^. The possibility of using hydrogenated Dowex 50WX8 resin for the recovery and separation of Pr(III), Dy(III) and Y(III) from aqueous nitrate solutions were carried out by Masry and coworkers and promising results were obtained in determined optimal operating conditions^28^.

The results of other reported works for the uses of SIR with different inert resin with other extractants have appeared promising in the absorption and separation of metals. TVEX-TOPO, which is a commercial low-cost macroporous copolymer resin containing 50% (w% Extractant fraction of total adsorbent mass) trioctylphosphine oxide (TOPO) was used for solid–liquid extraction of tungsten(VI) (W(VI)) and molybdenum(VI) (Mo(VI)) from nitric acid^29^. Sorption of molybdenum (VI) from nitric acid solution by solvent impregnated resin (SIR) technique using bio-beads styrene divinyl benzene copolymer (SM-4) impregnated with tri-alkyl phosphine oxide (CYANEX 923 or CY 923) extractant in both batch and column systems was studied and the obtained results indicate the selectivity of the technique for molybdenum recovery from nitric acid solution^30^.

The extraction of Pb(ΙΙ) nitrate solution by (AXAD-7) resin impregnated with organophosphorus extractants (DEHPA, IONQUEST, CYANEX-272) was investigated by Draa et al.^31^. The data revealed that with improving the pH of the aqueous solution, the sorption efficiency of metal ions increases. The highest sorption efficiencies of Pb(ΙΙ) ions using DEHPA and IONQUEST extractants are 95% in the pH range of 2.75–3 and 3.5–3.75, correspondingly.

El-Sofany^32^ evaluated the removal of La(ΙIΙ) and Ga(ΙΙΙ) from nitrate medium by (AXAD-7) resin impregnated via Aliquat-334 extractant. According to their reports, the experimental adsorption data for La(ΙIΙ) and Ga(ΙΙΙ) fit better with the Freundlich isotherm, and the highest adsorption capacity for La(ΙIΙ) and Ga(ΙΙΙ) using impregnated resin is 4.73 and 4.4 mg/g, respectively.

Belkholuch and Didi^33^ investigated the removal of Bi(III) ions from aqueous nitrate solution through AXAD-1180 impregnated with D2EHPA. Based on the obtained results, the equilibrium condition for bismuth ions adsorption is attained after 30 min. The maximum extraction yield (98.5%) of bismuth ions from mixture with the primary concentration of 250 mg/L at pH 3.6 was obtained using 15 mmol/g SIR.

Fouad et al.^34^ employed alizarin red S-impregnated XAD-2010 as an adsorbent for pre-concentration and separation of uranium and thorium in their bearing rocks. They reported the highest adsorption amount of 18.25 mg/g and 20.2 mg/g for Th(IV) and U(VI) ions, correspondingly. According to the Langmuir model, alizarin red S-impregnated XAD-2010 is considered an effective adsorbent for U(VI) and Th(IV) recovery.

Liao et al.^35^ examined the removal mechanism of heavy rare earth elements from hydrochloric acid solution by CYANEX-272-p507 impregnated resin. According to the results, the molar ratio of Cyanex-272-P507 to HREE (heavy rare earth elements) in the extraction complex is 3.

Kinetic studies of the divalent cadmium ions extraction with impregnated resins organized by adsorption of di(2-ethylhexyl) phosphoric acid onto macro-porous polymeric support of (AXAD-7) resin were performed by Benamor et al.^36^ using the homogenous diffusion model. According to the obtained results, a cadmium diffusion coefficient of about 10^−12^ m^2^/s was predicted in the range of investigated parameters.

Chen et al.^37^ investigated the optional separation of Vanadium from Molybdenum ions via the impregnation technique including blends of organophosphorus extractant (DEHPA) complexes stabled onto AXAD-4. The findings of this study revealed that the D2EHPA- Immobilized AXAD -4 resin adsorbs Molybdenum ions higher than Vanadium ions, and the best separation of these ions occurs at pH 6.

The Adsorption of U(VΙ) ions from Egyptian crude phosphoric acid through a batch experiment method using AXAD -2 resin fertilized with tri-butyl phosphate (TBP) and di-2-ethylhexyl phosphoric acid (D2EHPA) as adsorbent was studied^38^. In the current work, the highest sorption capacity of 67 mg/g was obtained for the adsorption of U(VI) ions on improved AXAD-2 resin. The data showed that the modified resin is an efficient adsorbent for U (VI) elimination from Egyptian crude phosphoric acid.

Sett et al.^22^ investigated the sorption performance of a group of rare earth elements on DEHPA-impregnated XAD-7 resin in a nitric acid medium. They stated that the adsorption process of rare earth elements reaches equilibrium after 120 min, and the Pseudo-Second-Order kinetic scheme is compatible with the experimental result.

Equilibrium and kinetics for the adsorption Biphenol-A from aqueous solutions with AXAD-7 resin containing Aliquat-336 have been studied in batch and column experiments by Betra et al.^39^. They assumed that the adsorbent with 1 g of Aliquat-336 per g of A XAD-7 was able to remove 88.98% of Biphenol-A from aqueous solutions. Also, the used IX7-1 resin was recovered 5 times and showed high stability during 5 adsorption–desorption cycles.

Moreover, a di(2-ethylhexyle) phosphoric acid (D2EHPA) fertilizedXAD-4 resin is produced and its adsorption–desorption performance was investigated for uptake of Sr(ΙΙ) ions in aqueous solutions^40^. According to the obtained results, the percentage adsorption of strontium ions onto the impregnated XAD-4 resin at optimal pH was 94.0%.

The literature review indicates that SIRs are efficiently employed for the extraction of metal ions in analytical and environmental applications. Easy handling, quick operation, high loading capacity as well as stability during several adsorption–desorption cycles, make this approach an efficient, economical, and promising technology for metal ions removal^41–45^.

The current work investigated the ability of AXAD-7 resin impregnated with CYANEX-272 (SIR) for Ce(ΙΙΙ) and La(ΙΙΙ) ions adsorption from aqueous solutions. The adsorption process modeling was performed using RSM according to CCD to assess the impact of pH of the metal ion solution, SIR dose, and temperature on Ce(ΙΙΙ) and La(ΙΙΙ) ions percentage adsorption. The sorption of Ce(ΙΙΙ) and La(ΙΙΙ) ions onto SIR also was assessed according to the equilibrium and kinetics. The isotherm approaches such as Langmuir, Freundlich, Temkin, and Sips were utilized to explain the equilibrium result. The kinetic result achieved from the batch sorption runs has been matched by pseudo-first-order, second-order correlations, and (IDMs).

## Materials and method

### Chemicals and tools

CYANEX-272 (di-2,4,4-trimethylpentylphosphinic acid), toluene (C_6_H_5_CH_3_), sodium hydroxide (NaOH), nitric acid (HNO_3_), lanthanum nitrate (La(NO_3_)_3_.6H_2_O), cerium nitrate (Ce(NO_3_)_3_.H_2_O) salts were of fluka products. Amberlite XAD-7 was prepared from Merck. All chemicals applied in this study were of analytical grade.

Ce(ΙΙΙ) and La(ΙΙΙ) ions measurements were done using PerkinElmer Optima 2000 DV Inductivity coupled plasma device atomic emission spectrometer (ICP-AES). The batch sorption tests were accomplished via a thermostatic shaking water bath GFL-1083 Model. A pH meter is used to calculate the solution pH (Metrohm 780 Model).

To characterize the prepared SIR, the fourier transform infrared (FTIR) spectrometer (Vector22-Bruker Company, Germany) at wavenumbers between 400 and 4000 cm^−1^ was used to recognize the resin functional groups. Specific surface area, pore size distribution, and microporosity of SIR have been specified by Brunauer–Emmett–Teller (BET) analysis using nitrogen as carrier gas at 77 K (Quantachrome Autosorb-1 model). The scanning electron microscope (SEM) combined with Energy Dispersive X-ray (EDX) using a TESCAN Vega TS 5136LM generally at acceleration voltage 20 kV, was employed to determine morphology and elemental constituents of prepared SIR.

### Preparation of the SIR

Before fertilization, the AXAD-7 resin was rinsed various times via distilled water to eliminate contaminations and then dehydrated at room temperature (25 °C ± 1).

The SIR was prepared by dry method; the impregnation solution was produced by mixing 0.87 g of CYANEX-272 in 30 ml toluene and mixed with 15 g of AXAD-7 resin. The resulting slurry was gently agitated for 3 h and then, the SIR was mixed with 0.1 mol L^−1^ Hydrochloric acid and dried at ambient room temperature (25 °C ± 1) for 48 h.

### RSM experimental plan

In the present study, CCD is employed to calculate the main interaction of 3 individual parameters including the primary solution pH (A), temperature (B), and SIR dose (C), on the adsorption of Ce(ΙΙΙ) and La(ΙΙΙ) aqueous solutions. In CCD, five levels are considered for each independent parameter, which includes a central point and two factorial points that are ± 1 unit away from the central point. There are also two-star points that make it possible to estimate the curvature and are ± α until away from the center point. Table 1 provided the coded and actual values of the independent factors.

**Table 1:** Coded and actual levels of the independent parameters in CCD (α = axial points).

Variable	Symbols	Levels				
		− α = − 2	− 1	0	+ 1	+ α = + 2
pH	A	2	3	4	5	6
Temperature (°C)	B	15	25	35	45	55
SIR dose (gr)	C	0.05	0.2	0.35	0.5	0.65

To investigate the percentage adsorption of Ce(ΙΙΙ) and La(ΙΙΙ) ions onto A XAD-7 resin fertilized with CYANEX-272, 20 experiments containing six duplicates at the midpoints (to assess the pure error) were performed. The experimental plan network is presented in Table 2.

**Table 2:** Experimental plan network and average calculated percentage adsorption of Ce(ΙΙΙ) and La(ΙΙΙ) ions.

Run order	Independent variables			Adsorption (%)			
	Initial pH	Sorption Temperature (°C)	SIR dose (g)	Ce(ΙΙΙ)		La(ΙΙΙ)	
				Experimental	Predicted	Experimental	Predicted
1	5	25	0.5	98.99	97.50	73.16	68.35
2	5	45	0.2	47.11	48.54	30.39	30.54
3	3	45	0.2	37.93	36.40	19.71	19.75
4	4	35	0.35	74.23	72.42	43.57	43.96
5	5	25	0.2	47.48	45.96	27.03	28.03
6	4	15	0.35	71.82	71.87	45.05	41.38
7	4	35	0.35	74.30	72.42	43.64	43.96
8	5	45	0.5	99.54	98.07	71.38	71.07
9	4	35	0.35	73.91	72.42	43.84	43.96
10	3	45	0.5	96.93	96.40	58.71	59.46
11	2	35	0.35	67.03	66.79	32.47	32.45
12	4	35	0.05	11.19	10.64	7.47	7.55
13	4	55	0.35	76.32	75.80	46.02	46.57
14	4	35	0.35	74.17	72.42	43.56	43.96
15	3	25	0.2	35.56	34.02	17.57	17.78
16	4	35	0.65	99.90	102.87	87.18	80.78
17	6	35	0.35	86.34	84.72	55.05	56.16
18	4	35	0.35	74.25	72.42	43.37	43.96
19	3	25	0.5	92.04	95.59	62.02	57.03
20	4	35	0.35	73.81	72.42	43.79	43.96

The output responses of Ce(ΙΙΙ) and La(ΙΙΙ) ions' percentage adsorption can be predicted using the quadratic model according to Eq. (1).

1 $$\mathrm{Y}={\upbeta }_{0}+\sum {\upbeta }_{\mathrm{i}}{\mathrm{x}}_{\mathrm{i}+}\sum {\upbeta }_{\mathrm{ii}}{\mathrm{x}}_{\mathrm{i}}^{2}+\sum \sum {\upbeta }_{\mathrm{ij}}{\mathrm{x}}_{\mathrm{i}}{\mathrm{x}}_{\mathrm{j}}$$

The above formula shows the predicted response with Y and the coefficients of the equation with ${\upbeta }_{\mathrm{ij}}{ ,\upbeta }_{\mathrm{ii}}{,\upbeta }_{\mathrm{i}}{,\upbeta }_{0}$ and the independent variables with X_i_ and X_j_ (i ≠ j). Independent variables are used in calculations in the form of coded rolling. The reliability of this model was explained based on the coefficients of determination (R^2^), and its adequacy was extra calculated by analysis of variance (ANOVA) and lack-of-fit test ^46–54^.

### Batch adsorption experiments

Simultaneous adsorption of Ce(ΙΙΙ) and La(ΙΙΙ) ions in the batch system was studied according to pH, SIR dose, and process temperature according to the experimental design matrix (Table 2). To perform adsorption experiments, the first 20 ml of a solution containing Ce(ΙΙΙ) and La(ΙΙΙ) ions with a specified concentration was poured into 100 ml polyethylene containers and the pH of the solution was regulated via nitric (HNO_3_) acid and (NaOH) of 0.1 M. Then a certain amount of resin was introduced to the mixture and the polyethylene containers were located in a shaker for 180 min at a certain temperature. After the desired time, the resins were isolated from the mixture through filter paper, and the equilibrium concentration of Ce(ΙΙΙ) and La(ΙΙΙ) ions in the solution was measured by ICP-AES. All tests were repeated twice and mean values were reported.

The adsorption capacity (q) of Ce(ΙΙΙ) and La(ΙΙΙ) per unit mass of resin (mg g^−1^) and the percentage adsorption of these metal ions from aqueous mixture were measured via Eqs. (2) and (3), respectively.

2 $$q = \left( {c_{i} - c_{e} } \right) \times \frac{V}{M}$$

3 $$\mathrm{\% Adsorption }= \frac{{c}_{i}-{c}_{e}}{ci}\times 100$$

In the above equations, C_i_(mg L^−1^) and C_e_(mg L^−1^) are the primary concentration and equilibrium concentration of metal ions in the solution, respectively^55^. The weight of the dry adsorbent is also denoted by M (g) and the volume of the solution by V (L).

### Adsorption/desorption studies

One of the important factors in selecting the adsorbent is the ability of the adsorbent to recover. If it is not possible to recover the adsorbent after the adsorption process, the adsorbent itself is a biological contaminant. To create the highest amount of adsorption/desorption cycles of Ce(ΙΙΙ) and La(ΙΙΙ) ions were adsorbed and desorbed till the adsorption is not anymore achievable. For this purpose, desorption was performed by mixing 1 g of the SIR involving adsorbed ions with 10 mL of 2 mol L^−1^ HCl mixture. The mixture was agitated for 24 h at 150 rpm at 25 C. Then, the SIR was isolated via filter paper and washed with purified water, and dehydrated at ambient conditions. This stage was duplicated till the Ce(ΙΙΙ) and La(ΙΙΙ) ions are irretrievably mounted to the adsorbent material shell, setting up the plan for the highest quantity of usage cycles.

## Results and discussion

### Characterization of the SIR

#### Infrared spectra analysis

**Figure 1:**
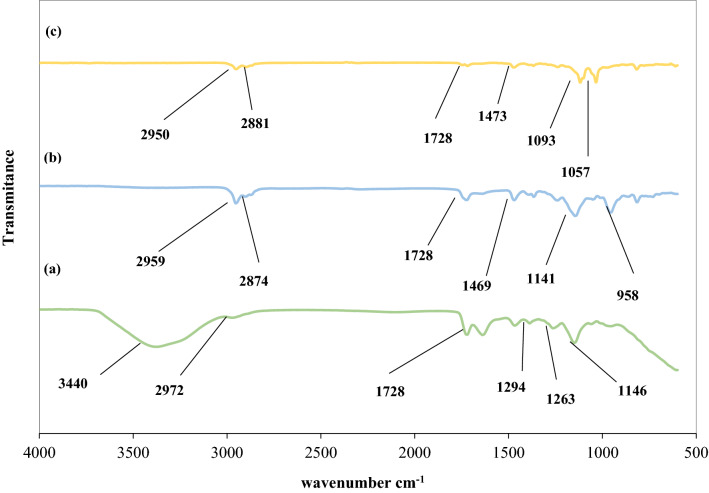
FTIR spectra of the working polymeric resin: (**a**) A XAD-7; (**b**) Amberlite XAD-7 resin impregnated with CYANEX-272; (**c**) AXAD-7 resin impregnated with CYANEX-272 after Ce(III) and La(III) adsorption.

FTIR spectra of AXAD-7 resin, CYANEX-272 fertilized resin, and Ce(ΙΙΙ) and La(ΙΙΙ) loaded resin was shown in Fig. 1a,b,c, correspondingly. As can be observed the band located in the limited area of 3500–3000 cm^-1^ is attributed to the presence of O–H bonds and is due to the presence of moisture in the Amberlite XAD-7 resin. After resin impregnation, the peak attributed to O–H bonds is lost, which is due to the disappearance of the hydrophilic nature of the SIR^22,39^.

The existence of the peaks placed at 2972 cm^−1^ could be accompanied with the stretching vibration of the aliphatic C–H group. The peak around 1728 cm^−1^ is attributed to C=O vibration which is present in both pure and impregnated XAD‒7 resins. The C–O stretching in the ester group can be observed near 1146 cm^−1^ and 1294 cm^−1^ location.

The peak located at 1469 cm^−1^ is attributed to the C-H deformation of the CH_3_ group observed in both pure and impregnated XAD‒7 resins^56^.

As shown in Fig. 1b the IR peaks about 2874 cm^−1^ and 2959 cm^−1^ are corresponding to CH_2_. The presence of P=O and P–O–H stretching vibration bonds located at 958 cm^−1^ and 1141 cm^−1^ can certify the successful impregnation of XAD‒7 resin with organophosphorus acid-specific groups^57^. After Ce(ΙΙΙ) and La(ΙΙΙ) adsorption, the intensity of specific peaks has reduced and subtle shifts in the wavenumber of many bands can be observed.

#### SEM–EDX analysis

**Figure 2:**
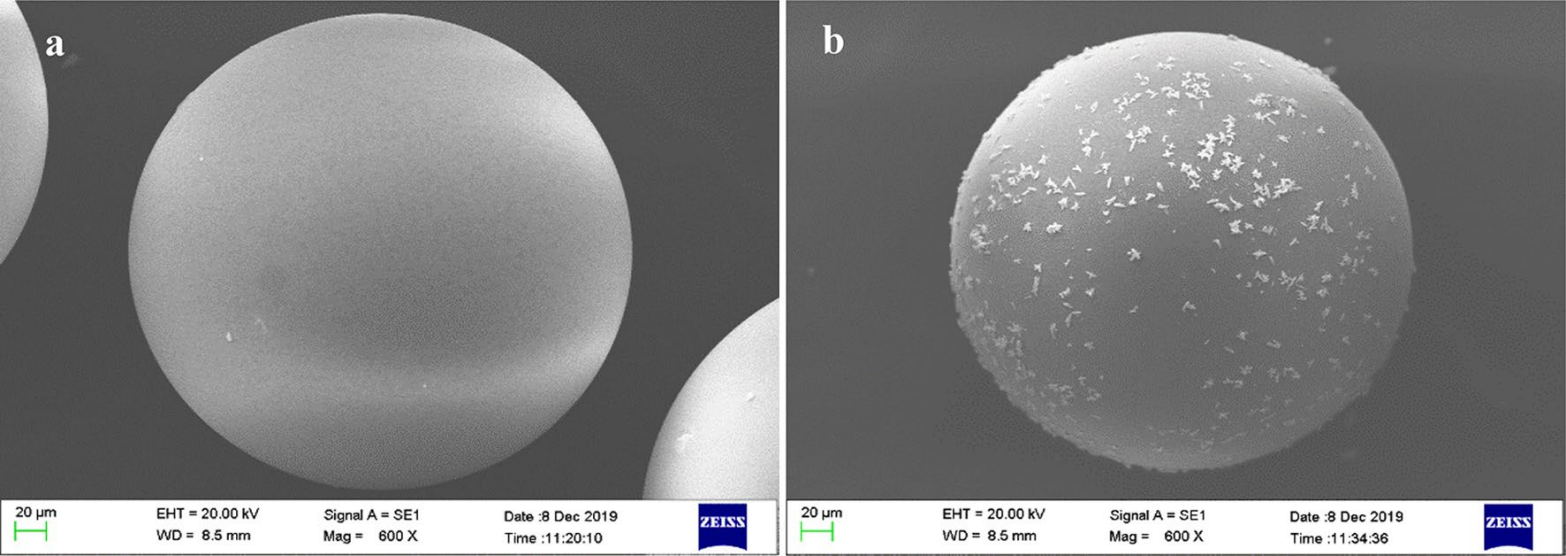
SEM image of A XAD-7 resin surface before (**a**) and after (**b**) impregnation with CYANEX-272.

The SEM analysis was performed to identify the shape and morphology of the outer surface of the XAD‒7 resin before and after impregnation with CYANEX-272 extractant (Fig. 2). As can be observed, the pure XAD‒7 resin is transparent, but it becomes cloudy after impregnation. This color change indicates the successful performance of the resin impregnation. As shown in Fig. 2b, changes also have been made on the outer surface of the resin after impregnation that affects the morphology of the resin. The presence of these micro granules fixed on the surface of XAD-7 resin indicates that it is functionalized by the CYANEX-272 extractant.

**Figure 3:**
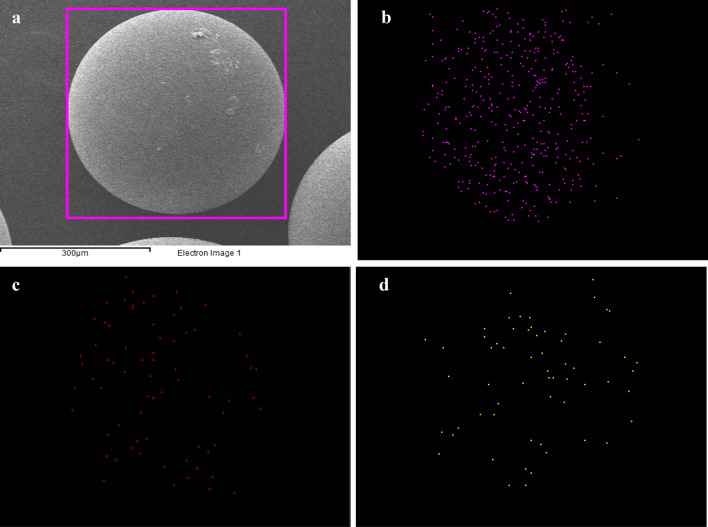
SEM pictures of the loaded SIR (**a**) and corresponding *p* (**b**), La (**c**) and Ce (**d**) elemental mappings.

The SEM images of the SIR after Ce(ΙΙΙ) and La(ΙΙΙ) adsorption and corresponding p, La, and Ce elemental mappings are presented in Fig. 3. As observed a large amount of phosphorus was diffused on the surface of XAD-7 resin after impregnation with CYANEX-272 extractant. The homogeneous distribution of La and Ce ions on the SIR surface confirms their successful adsorption by impregnated XAD-7 resin^22^.

**Figure 4:**
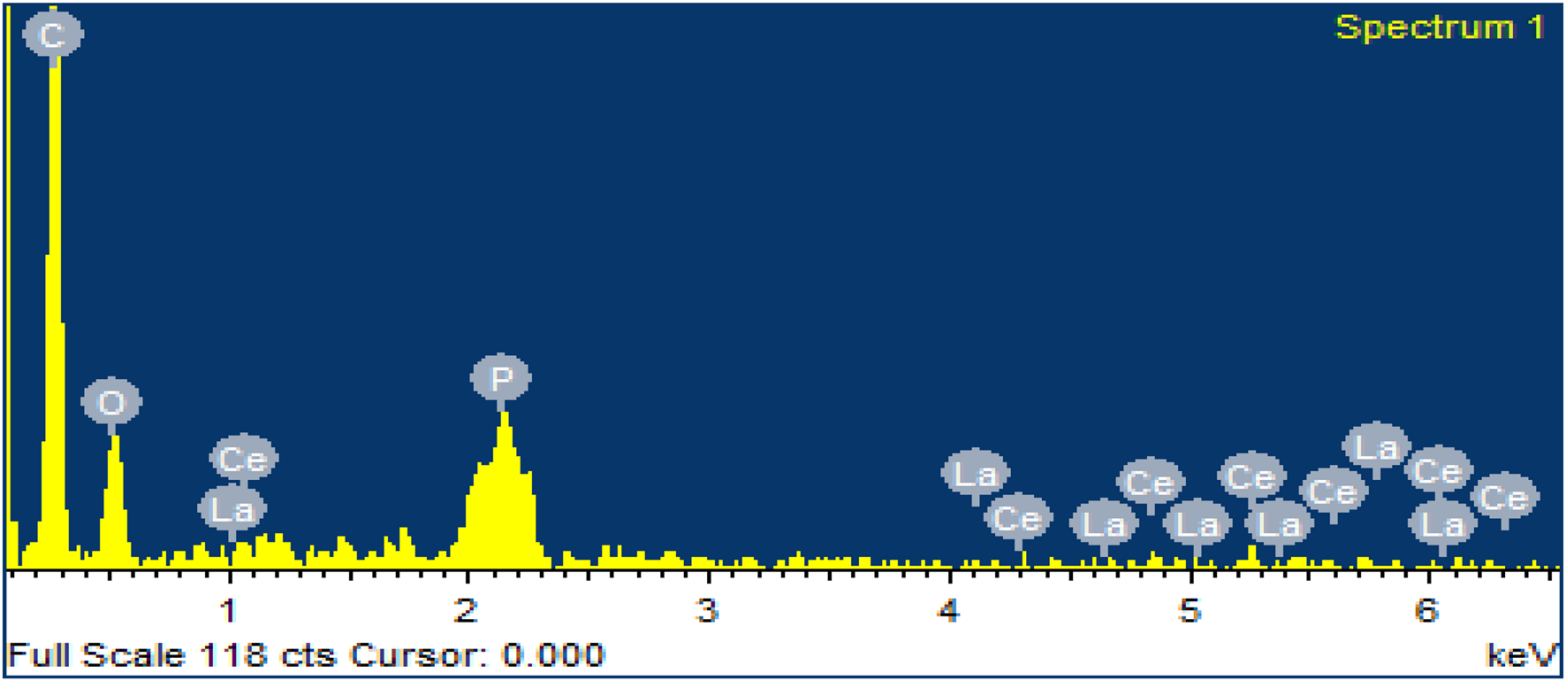
EDX image of SIR after Ce(ΙΙΙ) and La(ΙΙΙ) ions adsorption.

The EDX image of the XAD-7 resin impregnated with CYANEX-272 after Ce(ΙΙΙ) and La(ΙΙΙ) adsorption is shown in Fig. 4. According to Fig. 4, the existence of the phosphorus (P) peak denotes the successful impregnation of the resin by the CYANEX-272 extractant. Also, the presence of Ce(ΙΙΙ) and La(ΙΙΙ) peaks indicates their uptake by the resin.

#### BET analysis

Table 3 presented the results of surface area and porosity analysis of A XAD-7 resin before and after impregnation with CYANEX-272. XAD-7 resin size 0.3–0.85 mm was used as support. According to the obtained results, BET Surface area and BJH adsorption average pore volume of Amberlite XAD-7 resin decreases after the impregnation process, which is due to the filling of the resin pores by CYANEX-272 organic molecules. In addition, the increase in the pore diameter of SIR indicates that in the impregnation process, first the smaller pores and then the larger pores are filled with CYANEX-272 organic molecules. The surface area and porosity analysis showed the successful saturation of the Amberlite XAD-7 resin by CYANEX-272^22^.

**Table 3:** Surface properties of Amberlite XAD‒7 and CYANEX-272 impregnated Amberlite XAD‒7.

Resin	BET surface area (m^2^/g)	BJH Adsorption average pore volume (cm^3^ g^−1^)	Adsorption Pore Diameter (Å)
Amberlite XAD-7	450	1.14	85.63
Amberlite XAD-7 + CYANEX-272	67.16	0.57	154.13

### Preliminary evaluation of the impregnation process

Preliminary experiments performed for the adsorption of Ce(ΙΙΙ) and La(ΙΙΙ) using pure Amberlite XAD-7 resin showed that the Ce(ΙΙΙ) and La(ΙΙΙ) percentage adsorption on the XAD-7 resin is very low. To investigate the impact of the impregnation process on the XAD-7 resin adsorption rate, Ce(ΙΙΙ) and La(ΙΙΙ) adsorption tests were performed using SIR. According to the results presented in Table 4, the percentage adsorption of Ce(ΙΙΙ) and La(ΙΙΙ) on SIR was significantly increased and this indicates the positive effect of CYANEX-272 impregnation on XAD-7 resin ability for Ce(ΙΙΙ) and La(ΙΙΙ) ions adsorption.

**Table 4:** Investigating the role of CYANEX-272 impregnation on the adsorption process (Adsorption conditions: [Ce -La] = 200 mg/L, pH = 5, resin dose = 0.4 g, agitation time: 24 h, Temperature: 25 °C).

Resin	Ce(IΙI) Adsorption (%)	La(ΙIΙ) Adsorption (%)
Amberlite XAD-7	25.75	21.93
Amberlite XAD-7 + CYANEX-272	95.41	67.14

### Statistical analysis

The process of Ce(ΙΙΙ) and La(ΙΙΙ) adsorption on SIR was improved via RSM according to CCD. For this purpose, 20 designed experiments were accomplished and the results are presented in Table 2. The (ANOVA) was conducted to evaluate the Ce(ΙΙΙ) and La(ΙΙΙ) percentage adsorption (Table 5).

**Table 5:** ANOVA for quadratic model parameters.

Source	Ce(IΙΙ)					La(ΙΙΙ)				
	Sum of squares	DF	Mean of squares	F-value	*P*-value	Sum of squares	DF	Mean of squares	F-value	*P*-value
Model	54.94	9	6.10	414.80	< 0.0001	43.43	9	4.83	12,592.06	< 0.0001
A:pH	1.06	1	1.06	72.30	< 0.0001	3.23	1	3.23	8433.92	< 0.0001
B:Temperature	0.0526	1	0.0526	3.57	0.0880	0.1530	1	0.1530	399.27	< 0.0001
C:SIR dose	47.56	1	47.56	3231.46	< 0.0001	39.00	1	39.00	1.02E + 05	< 0.0001
AB	0.0001	1	0.0001	0.0056	0.9418	6.43E-06	1	6.43E-06	0.0168	0.8995
AC	0.3609	1	0.3609	24.52	0.0006	0.0658	1	0.0658	171.74	< 0.0001
BC	0.0127	1	0.0127	0.8619	0.3751	0.0024	1	0.0024	6.25	0.0315
A^2^	0.0500	1	0.0500	3.40	0.0952	0.0019	1	0.0019	4.96	0.0501
B^2^	0.0106	1	0.0106	0.7190	0.4163	3.31E-06	1	3.31E-06	0.0086	0.9278
C^2^	5.13	1	5.13	348.88	< 0.0001	0.9125	1	0.9125	2381.32	< 0.0001
Residual	0.1472	10	0.0147			0.0038	10	0.0004		
Lack of Fit	0.1227	5	0.0245	5.02	0.0506	0.0031	5	0.0006	4.32	0.0671
Pure Error	0.0245	5	0.0049			0.0007	5	0.0001		
Cor Total	55.09	19				43.43	19			
	R^2^		0.9973			0.9999				
	R^2^_Adj_		0.9949			0.9998				
	R^2^_pred_		0.9815			0.9994				
	Adeq.precision		80.3922			451.1744				
	C.V %		1.48			0.3025				

According to statistical analysis, the quadratic polynomial model with square root transform function was recommended to evaluate the investigated responses and recommended models for Ce(ΙΙΙ) and La(ΙΙΙ) ions percentage adsorption are presented in Eqs. (4) and (5), respectively.

4 $$\begin{aligned} {\text{Sqrt}}\left( {\% {\text{Ad Ce}}} \right) = & { 8}.{51} + 0.{\text{2579 A}} + 0.0{\text{573 B}} + {1}.{\text{72 C}} - 0.00{\text{32 AB}} - 0.{\text{2124 AC}} - 0.0{\text{398 BC}} \\ & + 0.0{\text{446 A}}^{{2}} + 0.0{2}0{\text{5 B}}^{{2}} - 0.{\text{4519 C}}^{{2}} \\ \end{aligned}$$

5 $$\begin{aligned} {\text{Sqrt}}\left( {\% {\text{Ad La}}} \right) = & { 6}.{63} + \, 0.{\text{4494 A}} + 0.0{\text{978 B}} + {1}.{\text{56 C}} + \, 0.000{\text{9 AB}} - \, 0.0{9}0{\text{7 AC}} - 0.0{\text{173 BC}} \\ & - 0.00{\text{87 A}}^{{2}} - 0.000{\text{4 B}}^{{2}} - 0.{19}0{\text{5 C}}^{{2}} \\ \end{aligned}$$

The mean squares are the result of dividing the total squares by the degree of freedom. The proportion of the mean squares of the results to the (MSE) represents the F-value index, which indicates the impact of each variable and their influences on the results. Its large size indicates the high impact of that variable on the response. The *p*-value index is applied to define the significance threshold of the four variables. Due to the 95% confidence interval, the importance of the variables per *p*-value is 0.05. As seen in Table 5, the F-values 414.80 and 12,592.06 for Ce(III) and La(III) accompanied by low probability quantities (*P* < 0.0001) show that the suggested patterns are considerable.

According to the ANOVA results R^2^ (coefficient of determination) were 0.997 and 0.999 for Ce(ΙΙΙ) and La(ΙΙΙ) adsorption models, respectively. In addition, R^2^_Adj_ (Adjusted R^2^) for Ce(ΙΙΙ) and La(ΙΙΙ) was equal to 0.995, which is close to 0.999, indicating the high accuracy of the model and it indicates that the model can make a good correlation between variables and the response.

Moreover, the signal-to-noise proportion described by the satisfactory accuracy were 80.39 and 451.17 for Ce(ΙΙΙ) and La(ΙΙΙ), respectively; The values of more than 4 for both elements indicates an adequate signal. The amount of data distribution relative to the mean is shown using the coefficient of variation (C.V %). Due to the C.V % values less than 10 for Ce(ΙΙΙ) (1.48) and La(ΙΙΙ) (0. 3025), the obtained models have good accuracy^58–62^.

**Figure 5:**
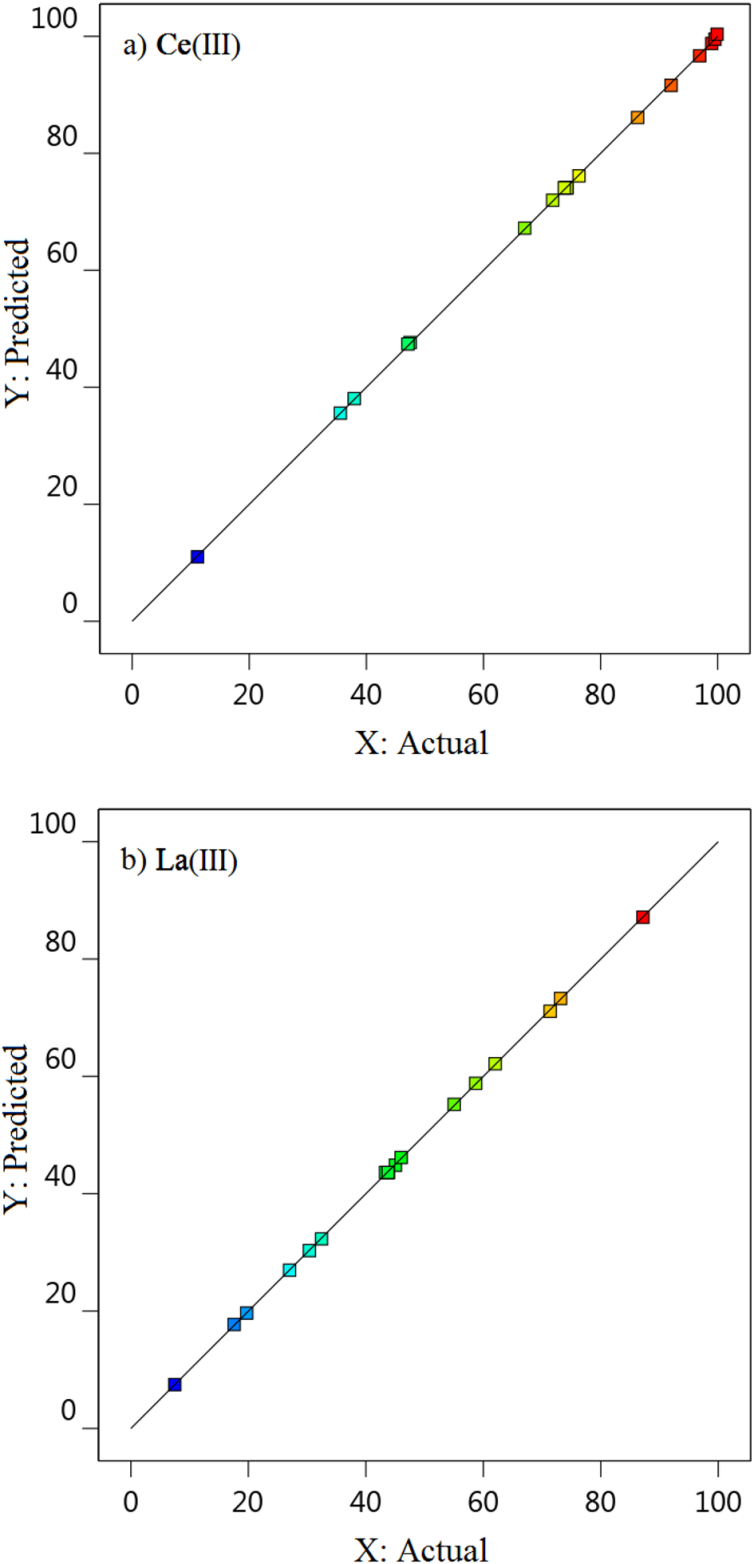
The sketch of calculated data versus experimental amounts of Ce(ΙΙΙ) and La(ΙΙΙ) ions adsorption.

The calculated F-values, *p*-values, (R^2^), and adjusted R^2^ (adj R^2^) are presented in Table 5. The data suggested that the recommended models were appropriate for the prediction of Ce(ΙΙΙ) and La(ΙΙΙ) ions percentage adsorption which is confirmed by a figure of estimated data versus experimental ones of Ce(ΙΙΙ) and La(ΙΙΙ) ions adsorption presented in Fig. 5^63^.

**Figure 6:**
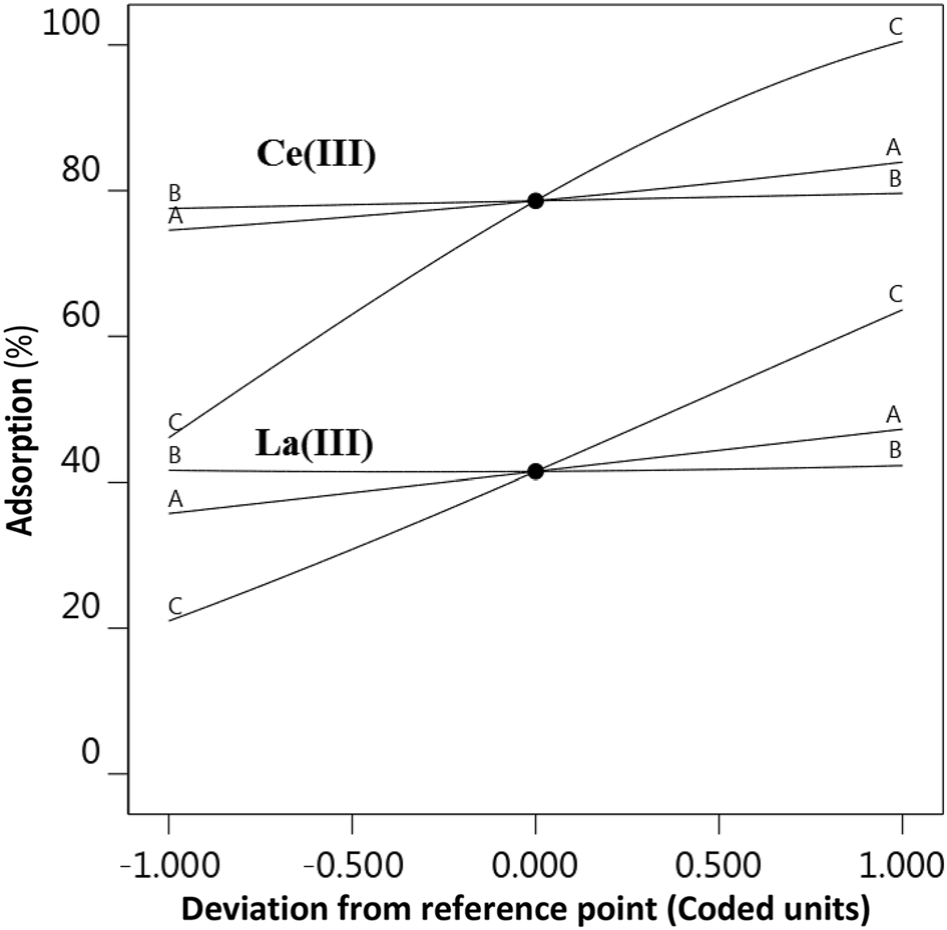
Perturbation plot for Ce(ΙΙΙ) and La(ΙΙΙ) ions adsorption models.

Perturbation plot for Ce(ΙΙΙ) and La(ΙΙΙ) ions adsorption models are presented in Fig. 6. As seen from this curve, the primary pH of the mixture (factor A), Temperature (factor B), and SIR dose (factor C) have a positive impact on Ce(ΙΙΙ) and La(ΙΙΙ) ions adsorption. According to the perturbation plot, the SIR dose was defined as the most effective parameter on Ce(ΙΙΙ) and La(ΙΙΙ) ions adsorption.

### Investigating the effects of process variables

To investigate the impact of independent factors and their relations on the investigated responses obtained by the polynomial models, 3D surface plots were prepared. 3D graphs show the superficial response of the performance of the two individual factors at the center part of the other individual variable on the percentage adsorption of Ce(III) and La(III) on the SIR.

**Figure 7:**
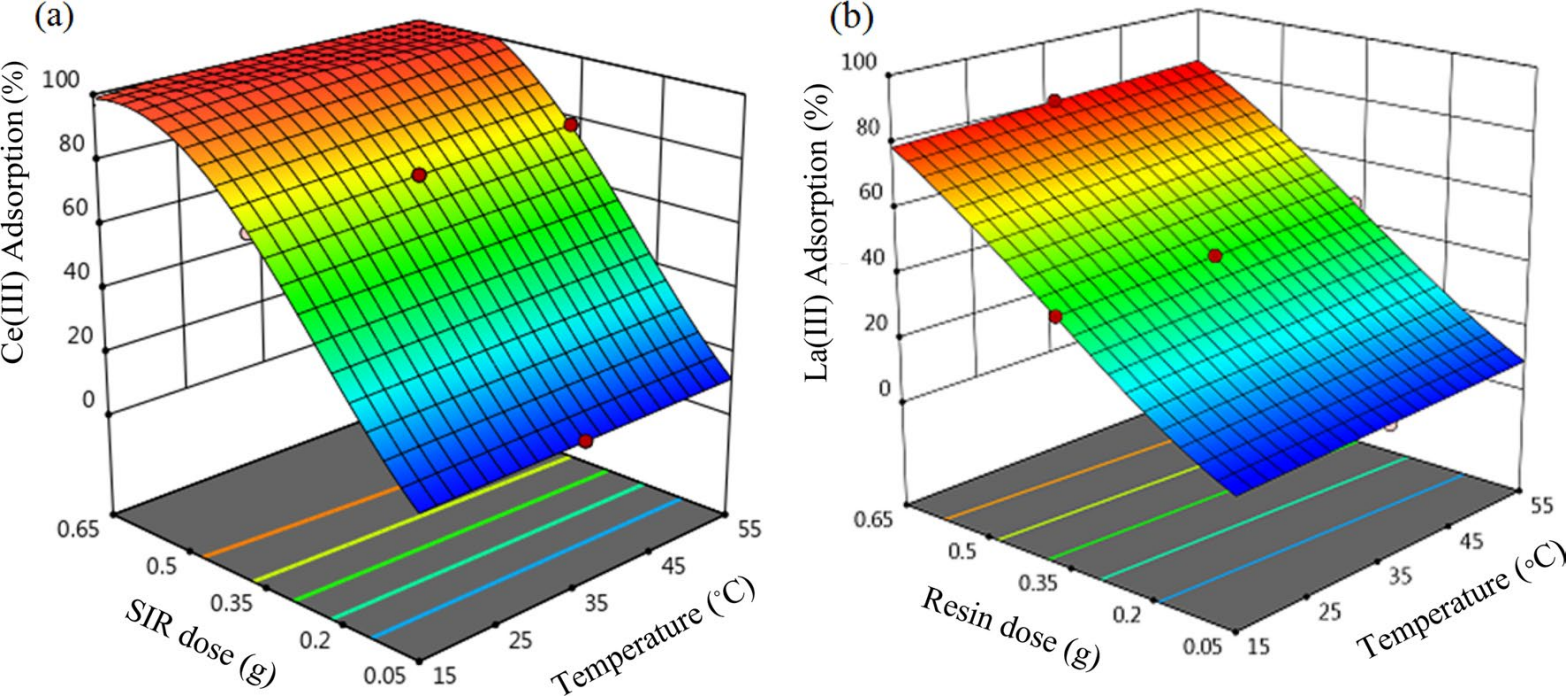
3D sketches of the combined impact of temperature and SIR dose on the percentage adsorption of (**a**) Ce(III) and (**b**) La(III) (primary metal ions concentration 200 mg L^−1^, pH = 4 and time 180 min).

Figure 7 represents the 3D plot, that illustrates the incorporated effect of temperature and SIR dose on the percentage adsorption of Ce(III) and La(III) at fixed pH of 4.

As can be seen from the graph, SIR dose significantly affects the percentage adsorption of Ce(III) and La(III) while with increasing temperature, the percentage adsorption of Ce(ΙΙΙ) and La(ΙΙΙ) ions slightly increases. By growing the SIR dose from 0.05 to 0.65 g at 35 °C, the percentage adsorption of Ce(ΙΙΙ) ions was increased from 11.19 to 99.90 and the percentage adsorption of La(ΙΙΙ) ions was increased from 7.47 to 87.18, respectively. This corresponds to the increment of the available adsorption sites for Ce(ΙΙΙ) and La(ΙΙΙ) ions.

**Figure 8:**
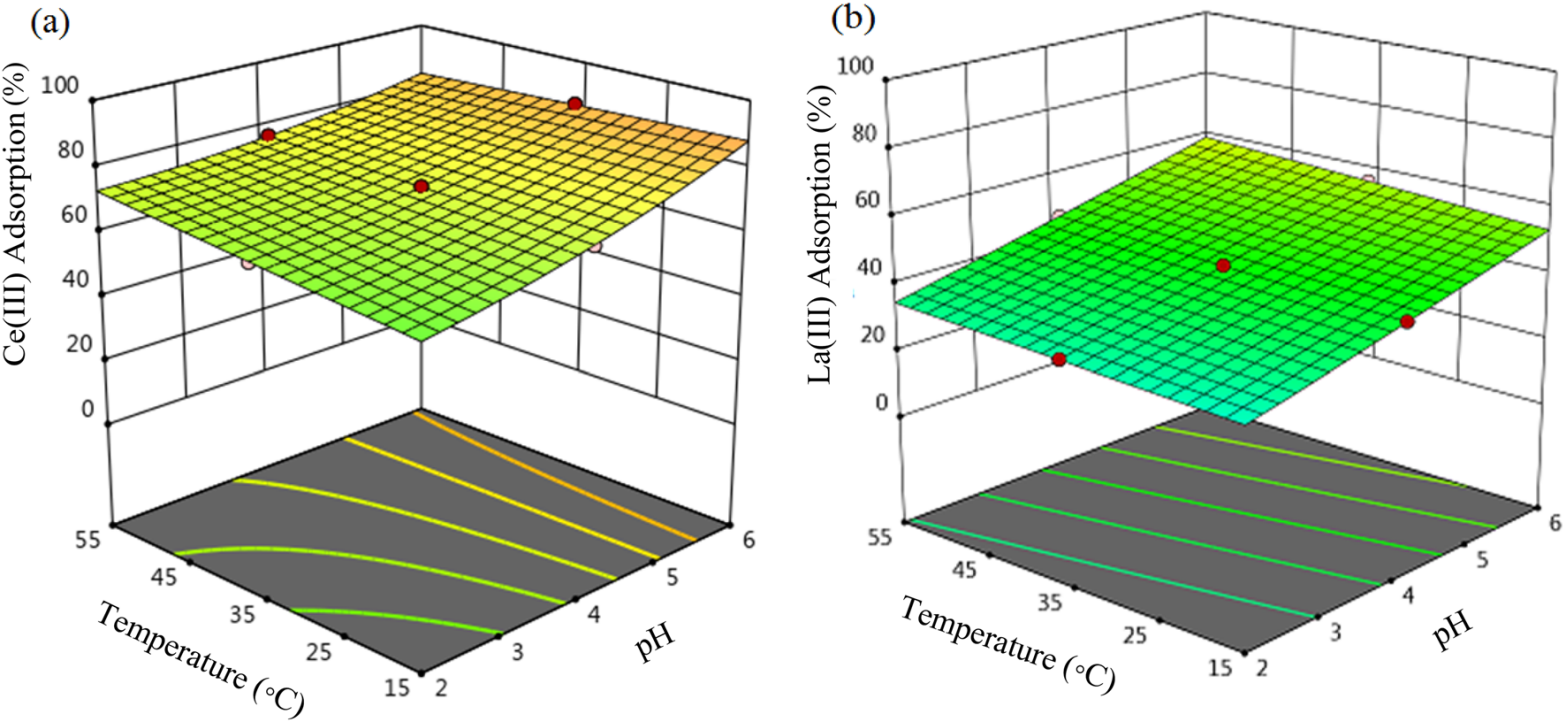
3D figures of the combined impact of pH and temperature on the percentage adsorption of (**a**) Ce(III) and (**b**) La(III) (primary metal ions concentration 200 mg L^−1^, SIR dose = 0.35 g and time 180 min).

The contemporaneous impact of primary mixture pH and temperature on the adsorption ratio of Ce(ΙΙΙ) and La(ΙΙΙ) using 0.35 g SIR is shown in Fig. 8.

As can be observed the impact of mixture pH on the adsorption percentage is greater than the temperature. By improving the primary solution pH from 2 to 6 at 35 °C, the percentage adsorption of Ce(ΙΙΙ) ions was increased from 67.03 to 86.34 and the percentage adsorption of La(ΙΙΙ) ions was increased from 32.47 to 55.05, respectively. Since the CYANEX-272 extractant used for impregnation of the resin is an acidic extractant and releases H^+^ during the adsorption process, at low pH H^+^ is more successful in competing with Ce(ΙΙΙ) and La(ΙΙΙ) cations in forming a complex with CYANEX-272 and the adsorption of Ce(ΙΙΙ) and La(ΙΙΙ) is low. With intensifying pH (decreasing the concentration of H_2_ ions)^32^, the amount of Ce(ΙΙΙ) and La(ΙΙΙ) ions adsorption improved and the highest adsorption occurs at pH 6. In this method, the surface of the resin is impregnated with a chelating solvent, usually containing the organic compounds of the cavity, and the ion exchange process is performed. Similar results have been obtained from other studies for the ion exchange mechanism in using impregnated Amberlite XAD7HP resin for the removal of Cd^2+^, Ni^2+^, Cu^2+^, and Pb^2+^ from aqueous solutions^64^. SIRs have the properties of ion exchange resins and solvent extraction methods simultaneously^19,23^.

**Figure 9:**
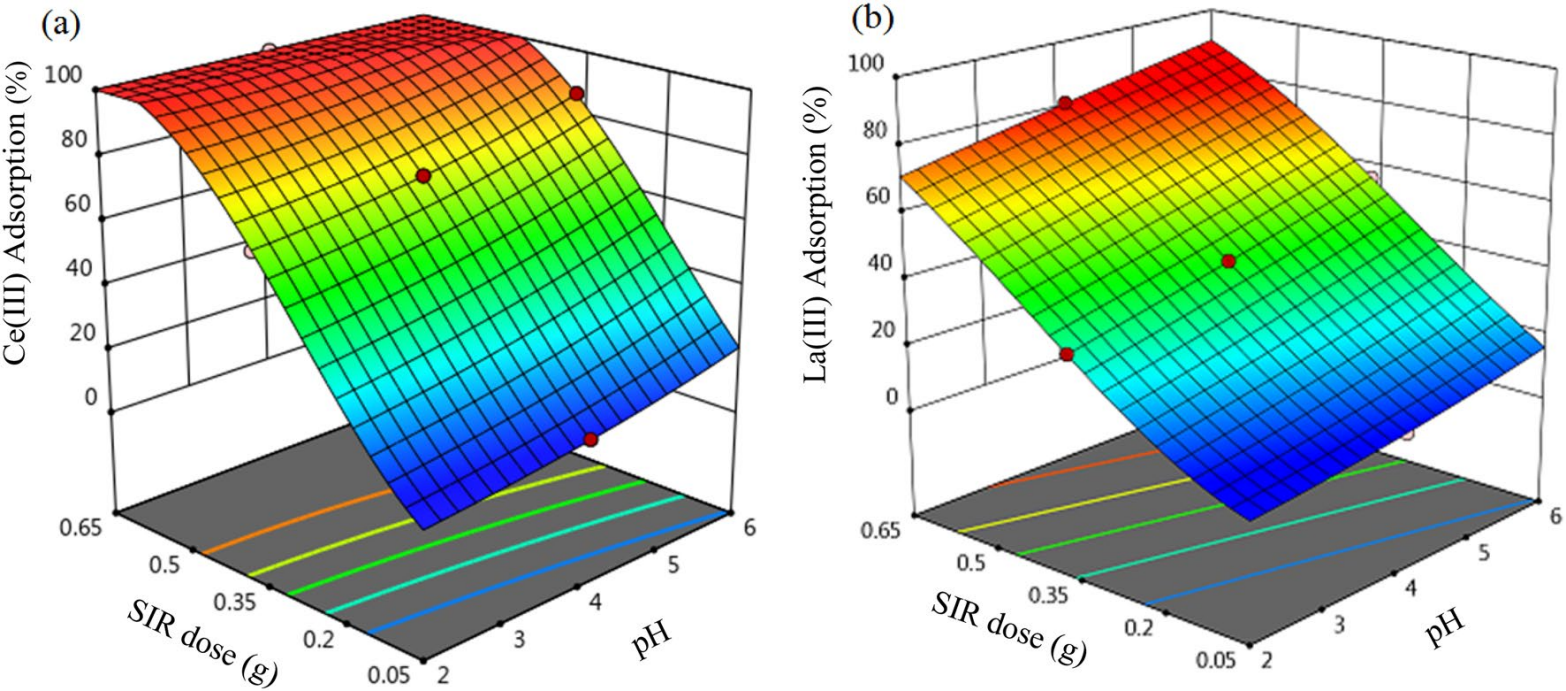
3D curves of the combined effect of pH and SIR dose on the percentage adsorption of (**a**) Ce(III) and (**b**) La(III) (primary metal ions concentration 200 mg L^−1^, temperature = 35 °C and time 180 min).

Figure 9 illustrates the combined impacts of primary mixture pH and SIR dose on Ce(ΙΙΙ) and La(ΙΙΙ) ions adsorption. According to the obtained results, the SIR dose has a greater effect on the percentage absorption of Ce(ΙΙΙ) and La(ΙΙΙ) ions than the primary solution pH. Investigation of the Ce(ΙΙΙ) and La(ΙΙΙ) ions adsorption process using SIR showed that the impact of SIR dose on the metal ions sorption is more than the two other independent variables (pH and temperature). The maximum percentage adsorption of Ce(ΙΙΙ) (100%) and La(ΙΙΙ) (97%) was achieved at pH of 6, SIR dose of 0.65 g and 35 °C.

According to Borai and coworkers, the normal Cerium precipitation process is highly dependent on the solution pH. This could be interpretated based on the following three equations that demonstrate the successive hydrolysis of Ce(III) in solution:

6 $${\text{Ce}}^{{{3} + }} \left( {{\text{aq}}} \right) + {\text{OH}}^{-} \left( {{\text{aq}}} \right) \leftrightarrow {\text{Ce}}\left( {{\text{OH}}} \right)^{{{2} + }} \left( {{\text{aq}}} \right)$$

7 $${\text{Ce}}\left( {{\text{OH}}} \right)^{{{2} + }} \left( {{\text{aq}}} \right) + {\text{OH}}^{-} \left( {{\text{aq}}} \right) \leftrightarrow {\text{Ce}}\left( {{\text{OH}}} \right)_{{2}}^{ + } \left( {{\text{aq}}} \right)$$

8 $${\text{Ce}}\left( {{\text{OH}}} \right)_{{2}}^{ + } \left( {{\text{aq}}} \right) + {\text{OH}}^{-} \left( {{\text{aq}}} \right) \leftrightarrow {\text{Ce}}\left( {{\text{OH}}} \right)_{{3}}$$

The obtained results showed that by further addition of OH^–^, the pH rises and Ce(III) precipitates out of solution after pH 7 as a cloudy white gellike precipitate, which is presumably Ce(OH)_3_(s)^65,66^. Regarding lanthanum, due to the fact that there is a possibility of precipitation in alkaline pHs^67^, therefore, the studied pH range was limited to acidic pHs.

### Reliability of the experimental models

To achieve optimum conditions for the highest percentage adsorption of Ce(ΙΙΙ) and La(ΙΙΙ) ions on SIR, the optimization process was performed using response surface methodology. For this purpose, the optimization goal was set on the maximum percentage adsorption of Ce(ΙΙΙ) and La(ΙΙΙ) ions using minimum SIR amount in the investigated primary solution pH and temperature range. The experimental situations which provided the highest desirability (pH 6, 0.45 g SIR, and 25 °C adsorption temperature) were nominated as the optimum condition and need to be verified. The predicted and experimental results of Ce(ΙΙΙ) and La(ΙΙΙ) ions percentage adsorption are presented in Table 6. According to the results, the attained experimental data were compatible with the estimated results from the models, with somewhat minor error quantities (< 1%).

**Table 6:** The optimum condition derived from RSM for Ce(III) and La(III) ions adsorption.

Metal ion	pH	Temperature (°C)	SIR dose (g)	Experimental	Predicted	Error %
Ce(III)	6	25	0.45	99.75	100	0.25
La(III)	6	25	0.45	69.98	70.67	0.98

### Investigating the adsorption kinetics

Investigation of adsorption kinetics is very useful for modeling the adsorption process and gives data regarding the adsorption mechanism and the transfer mode of solutes from the aqueous phase to the solid phase. The Ce(ΙΙΙ) and La(ΙΙΙ) adsorption kinetics were studied in the range of 5–240 min at a temperature of 25 °C, pH 6, the primary concentration of 200 mg L^−1^, and SIR dose of 0.35 g. The obtained experimental result was examined by means of pseudo-first-order, the pseudo-second-order, and (IDMs) in their nonlinear form.

The nonlinear procedure of pseudo-first-order, pseudo-second-order, and (IDMs) are expressed by Eqs. (9), (10) and (11), respectively.

9 $${\mathrm{q}}_{\mathrm{t}}=\left({\mathrm{q}}_{\mathrm{e}}-{\mathrm{e}}^{-{\mathrm{k}}_{1}\mathrm{t}}\right)$$

10 $${\mathrm{q}}_{\mathrm{t}}=\frac{{\mathrm{k}}_{2}{\mathrm{q}}_{\mathrm{e}}^{2}\mathrm{t}}{1+{\mathrm{k}}_{2}{\mathrm{q}}_{\mathrm{e}}\mathrm{t}}$$

11 $${\text{q}}_{{\text{t}}} = {\text{k}}_{{{\text{id}}}} {\text{t}}^{{{\raise0.7ex\hbox{$1$} \!\mathord{\left/ {\vphantom {1 2}}\right.\kern-\nulldelimiterspace} \!\lower0.7ex\hbox{$2$}}{\text{~}}}} + {\text{c}}$$

In these equations, q_e_ (mg g^−1^) is the adsorption capacity at equilibrium, and q_t_ (mg g^−1^) is the adsorption capacity at time t (min). The k_1_ (min^−1^) and k_2_ (g mg^−1^ min^−1^) are the pseudo-first-order and pseudo-second-order equation rate constants, correspondingly. In Eq. (8), K_P_ (mg g^−1^ min^−0.5^) and C denote the intraparticle diffusion rate constant and thickness of the boundary layer, respectively^68–71^.

The results of nonlinear kinetic data fitting for Ce(ΙΙΙ) and La(ΙΙΙ) ions adsorption on SIR are presented in Table 7. The amount of the kinetic models that matched the experimental results are evaluated using several error functions i.e. residual sum of squares error (SSE), coefficient of determination (R^2^), root mean square error (RMSE), nonlinear chi-square (χ^2^), and hybrid fractional error function (HYBRID). According to the reported results, the pseudo second-order kinetic model is well fitted to the experimental Ce(ΙΙΙ) and La(ΙΙΙ) ions adsorption data. This model relies on the supposition that chemisorption may be the rate-controlling step of the adsorption process and the Ce(ΙΙΙ) and La(ΙΙΙ) ions attached to the resin surface forms a chemical (in general covalent) bond and tends to find sites that make the coordination number the greatest possible with the surface^72–76^.

**Table 7:** Parameters of kinetic models for Ce(III) and La(III) Adsorption onto SIR ([Ce-La] = 200 mg/L, pH = 5, SIR dose = 0.4 g, Stirring speed = 200 rpm, and temperature = 25 ± 2 °C).

Pseudo first order	Values		Pseudo second order	Values		Intraparticle diffusion	Values	
	Ce(III)	La(III)		Ce(III)	La(III)		Ce(III)	La(III)
q_e_ (mg g^−1^)	10.068	6.292	q_e_ (mg g^−1^)	11.829	7.935	K_id_ (mg g^−1^ min^−0.5^)	0.659	0.456
k_1_ (min^−1^)	0.024	0.015	k_2_ (g mg^−1^ min^−1^)	0.0028	0.0017	C	1.621	− 0.083
R^2^	0.977	0.986	R^2^	0.993	0.994	R^2^	0.855	0.962
SSE	1.744	0.373	SSE	0.512	0.209	SSE	11.120	1.240
χ^2^	0.385	0.163	χ^2^	0.192	0.048	χ^2^	2.122	0.354
RMSE	0.467	0.216	RMSE	0.253	0.162	RMSE	1.179	0.394
HYBRID	− 9.116	− 11.383	HYBRID	− 0.055	− 0.227	HYBRID	0.441	− 0.105

**Figure 10:**
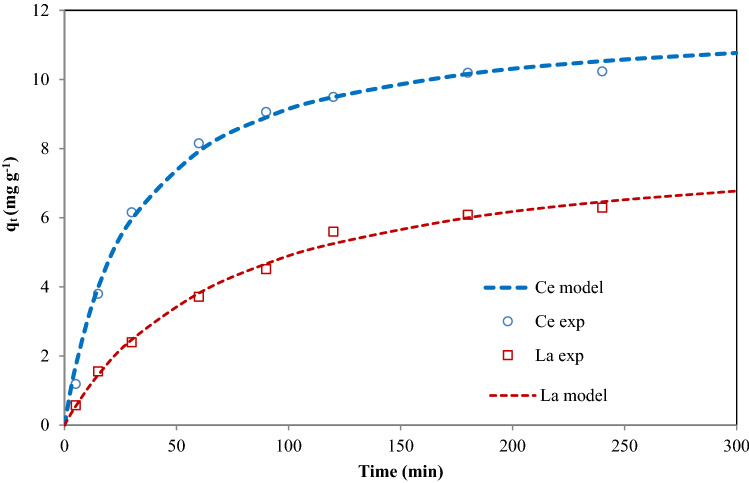
Nonlinear fit of the pseudo-second order kinetic model of Ce(III) and La(III) ions adsorption onto SIR ([Ce-La] = 200 mg/L, pH = 5, SIR dose = 0.4 g, Stirring velocity = 200 rpm, and temperature = 25 ± 2 °C).

According to the data depicted in Fig. 10, the adsorption capacity of Ce(ΙΙΙ) and La(ΙΙΙ) increased with rising contact time from 5 to 180 min and then remained constant due to the accumulation of Ce(ΙΙΙ) and La(ΙΙΙ) in the active sites on the SIR. Studies show that most of the adsorption process occurs in the first 80 min, which is due to the occupation of available resin surface sites for the adsorption of Ce(ΙΙΙ) and La(ΙΙΙ) ions. After all the foreign active sites on the SIR were saturated, the adsorption process slowed down to equilibrium. The reason for this is the low rate of saturation of the internal active sites of the adsorbent by metal ions and after 180 min the adsorption process reaches equilibrium.

The equilibrium adsorption capacity (q_e_) is found to be 11.829 mg g^−1^ and 7.935 mg g^−1^ for Ce(ΙΙΙ) and La(ΙΙΙ) ions, correspondingly. The rate constant of PSO equation (k_2_) of 0.0028 and 0.0017 g mg^−1^ min^−1^ is calculated for Ce(ΙΙΙ) and La(ΙΙΙ) ions, respectively. These results demonstrate that the PSO adsorption mechanism is overriding and the rate of each ion is controlled by the chemisorption process.

### Investigating the adsorption isotherms

Adsorption isotherms are mathematical relationships that aim to establish a quantitative correlation between the value of matter adsorbed on a solid surface and its concentration in the liquid phase. Equilibrium adsorption isotherm provides important information about the adsorption mechanism. In this study, 6 isotherms patterns of Langmuir, Freundlich, Temkin, and Sips were employed in nonlinear form for analyzing the Ce(ΙΙΙ) and La(ΙΙΙ) ions adsorption on SIR in the concentration limit of 50 to 250 mg L^−1^^51^. The nonlinear type of Freundlich, Langmuir, Temkin, and sips isotherm models are expressed by Eqs. (12)–(15), correspondingly.

12 $${\text{q}}_{{\text{e}}} = {\text{k}}_{{\text{f}}} {\text{C}}_{{\text{e}}}^{{1/n}}$$

13 $${\mathrm{q}}_{\mathrm{e}}=\frac{{{\mathrm{q}}_{\mathrm{m}}\mathrm{k}}_{\mathrm{L}}{\mathrm{C}}_{\mathrm{e}}}{(1+{\mathrm{k}}_{\mathrm{L}}{\mathrm{C}}_{\mathrm{e}})}$$

14 $${\mathrm{q}}_{\mathrm{e}}=\frac{\mathrm{RT}}{{\mathrm{b}}_{\mathrm{T}}}\mathrm{ln}({\mathrm{A}}_{\mathrm{T}}{\mathrm{C}}_{\mathrm{e}})$$

15 $${\mathrm{q}}_{\mathrm{e}}=\frac{{\mathrm{q}}_{\mathrm{m}}{\mathrm{K}}_{\mathrm{S}}{\mathrm{C}}_{\mathrm{e}}^{{\mathrm{m}}_{\mathrm{S}}}}{1+ {\mathrm{K}}_{\mathrm{S}}{\mathrm{C}}_{\mathrm{e}}^{{\mathrm{m}}_{\mathrm{S}}}}$$

where C_e_ is the metal ions equilibrium concentration (mg L^−1^), and q_e_ is the equilibrium adsorption value (mg g^−1^).

In the Freundlich equation, k_f_ signifies the adsorbent capacity (L g^−1^), and n reflects the intensity of the adsorption.

In the Langmuir isotherm pattern, q_m_ is the maximum adsorption capacity (mg g^−1^), and K_L_ signifies the Langmuir constant (L mg^−1^).

In Temkin isotherm, ${\mathrm{B}}_{\mathrm{T}}=\frac{\mathrm{RT}}{{\mathrm{b}}_{\mathrm{T}}}$ is the Temkin constant, and A_T_ is the equilibrium binding constant (L g^−1^).

In the Sips isotherm model, q_ms_ is the Sips maximum adsorption capacity (mg g^−1^) and K_S_ is the equilibrium constant (L mg^−1^), and ms is the Sips model exponent.

The parameters of the investigated isotherm models for Ce(III) and La(III) adsorption onto SIR were acquired using nonlinear least-squares fitting of the experimental data and presented in Table 8. The quality of investigated isotherm models was evaluated using previously mentioned error functions^77,78^.

**Table 8:** Isotherm models’ factors and assessed quantities for Ce(III) and La(III) adsorption onto SIR (pH = 5, SIR dose = 0.4 g, stirring speed = 200 rpm, contact time = 180 min, and temperature = 25 ± 2 °C).

Langmuir	Ce(III)	La(III)	Freundlich	Ce(III)	La(III)
q_m_ (mg g^−1^)	10.489	6.2436	K_f_ (mg g^−1^)(L mg^−1^)^−n^	5.588	4.174
K_L_(L mg^−1^)	1.854	5.063	n	5.533	10.532
R^2^	0.972	0.988	R^2^	0.936	0.801
SSE	1.630	0.121	SSE	2.969	1.873
χ^2^	0.517	0.020	χ^2^	0.532	0.454
RMSE	0.571	0.155	RMSE	0.771	0.612
HYBRID	− 14.080	− 0.413	HYBRID	4.798	1.559

Temkin	Ce(III)	La(III)	Sips	Ce(III)	La(III)
$\frac{1}{{\mathrm{b}}_{\mathrm{T}}}$(J mol^−1^)	5.453E−04	2.028E−04	q_m_ (mg g^−1^)	11.873	7.324
A_T_	79.146	4248.859	${{\mathrm{K}}_{\mathrm{S}}(\mathrm{L }{\mathrm{mg}}^{-1})}^{{\mathrm{m}}_{\mathrm{S}}}$	1.056	3.590
			${\mathrm{m}}_{\mathrm{S}}$	0.577	0.818
R^2^	0.989	0.866	R^2^	0.998	0.993
SSE	0.545	1.336	SSE	0.100	0.071
χ^2^	0.078	0.314	χ^2^	0.011	0.011
RMSE	0.330	0.517	RMSE	0.141	0.119
HYBRID	1.343	0.911	HYBRID	0.697	0.046

According to the obtained data, the Sips isotherm was the best fitting isotherm model for evaluating Ce(III) and La(III) ions adsorption onto SIR. The maximum adsorption capacity obtained for Ce(III) and La(III) ions were 11.873 and 6.324 mg g^−1^, respectively.

The 3-parameter Sips isotherm pattern is an association of Langmuir and Freundlich two-parameter patterns and therefore it could theoretically offer better calculations for equilibrium results than these two isotherm models. The sips isotherm model showed the monolayer adsorption of one adsorbate molecule onto 1/ns adsorption sites and is suitable for estimating the heterogeneous adsorption scheme. This model also overcomes the drawback associated with the Freundlich isotherm model when the concentration is satisfactorily high^79,80^.

### Investigating the desorption process

The literature review indicates that SIRs are efficiently employed for the extraction of metal ions with well stability during several adsorption–desorption cycles. In present work also, the used Amberlite XAD-7 resin impregnated via CYANEX-272 extractant was recovered 3 times and showed high stability during 3 adsorption–desorption cycles.

**Figure 11:**
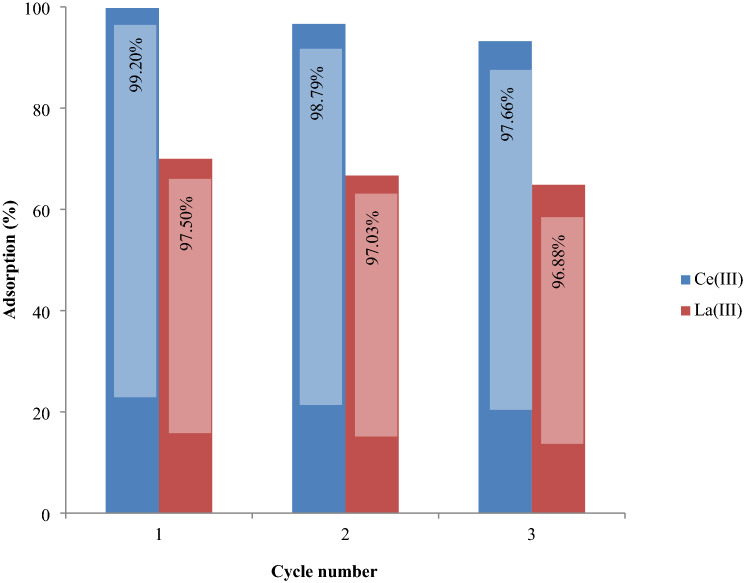
Adsorption–Desorption efficiency of SIR in 3 consecutive cycles using HCL 0.1 mol L^−1^.

The desorption study on the SIR containing Ce(ΙΙΙ) and La(ΙΙΙ) ions was conducted using 0.1 M HCl solution and the data are illustrated in Fig. 11. As could be observed, the percentage adsorption of Ce(III) and La(III) ions onto SIR decreased from 99.75% and 69.98% in the first cycle to 93.22% and 64.85% in the third cycle, respectively. Furthermore, the efficiency of 0.1 M HCl in the desorption of Ce(III) and La(III) ions from SIR decreased from 99.20% and 97.50% in the first cycle to 97.66% and 96.88% in the third cycle, correspondingly. As could be observed, the Ce(III) and La(III) ions recovery values are over 96%. According to the obtained results, SIR could be reprocessed many times with no substantial reduction in metal ion adsorption percentage. Similar results have been obtained from other studies for the regeneration and metallic ions recovery for using impregnated Amberlite XAD7HP resin for As(V), Pb(II) and Cd(II) sorption^81^.

## Conclusions

The current research work was performed to study the adsorption of Ce(ΙΙΙ) and La(ΙΙΙ) ions from an aqueous mixture through A XAD-7 resin impregnated with CYANEX-272 in the batch system. The prepared SIR was characterized using SEM–EDS, BET, and FTIR analysis techniques. The impact of three process-independent factors individual pH, Temperature, and SIR dose was scrutinized via RSM according to CCD. The Ce(ΙΙΙ) and La(ΙΙΙ) ions adsorption data were compatible to a second order polynomial model with the square root transform function. Based on the analysis of variance (ANOVA), the SIR dose was determined as the most effectual factor on Ce(ΙΙΙ) and La(ΙΙΙ) ions percentage adsorption. The equilibrium results were examined using the Langmuir, Freundlich, Temkin, and Sips sorption isotherm models. The data revealed the compromise of Ce(III) and La(III) ions adsorption onto SIR with the Sips isotherm model and the highest adsorption value obtained for Ce(III) and La(III) ions were 11.873 and 6.324 mg g^−1^, correspondingly. The kinetic studies were conducted using the pseudo-first-order, the pseudo-second-order, and (IDMs). based on the data obtained, kinetic data were fitted satisfactory to a pseudo-second-order rate correlation. The adsorption/desorption study was also performed for 3 cycles and the data revealed that the SIR could be reused several times for Ce(ΙΙΙ) and La(ΙΙΙ) ions adsorption without a significant performance decrement. According to the results, AXAD-7 resin impregnated with CYANEX-272 can be effectively used for Ce(ΙΙΙ) and La(ΙΙΙ) ions adsorption from aqueous solutions.

## Data Availability

The datasets used and/or analyzed during the current study are available from the corresponding author on reasonable request.
